# Genome-Wide Characterization of the *GRAS* Gene Family in Three Apiaceae Vegetables with Evolutionary Implications Across Representative Plants

**DOI:** 10.3390/life16071113

**Published:** 2026-07-03

**Authors:** Xiao Ma, Qiaoying Pei, Mengyao Shi, Xiaojie Li, Di Guo, Xinyao Zhang, Zipeng Meng, Rong Zhou, Yi Liang, Xiaoming Song

**Affiliations:** 1 Library/School of Life Sciences/School of Basic Medical Sciences, North China University of Science and Technology, Tangshan 063210, China; maxiao_1220@126.com (X.M.);; 2 National Engineering Research Center for Vegetables, Beijing Vegetable Research Center, Beijing Academy of Agriculture and Forestry Sciences, Beijing 100097, China; 3 Beijing Key Laboratory of Vegetable Germplasm Improvement, Beijing Academy of Agriculture and Forestry Sciences, Beijing 100097, China; 4 College of Horticulture, Nanjing Agricultural University, Nanjing 210095, China; 5 Department of Food Science, Aarhus University, 8200 Aarhus, Denmark

**Keywords:** *GRAS* transcription factors, Apiaceae vegetables, comparative genomic analysis, gene duplication/loss, co-expression networks, evolutionary origination

## Abstract

GRAS transcription factors are crucial regulators governing plant growth and stress adaptation, yet no systematic genome-wide investigation of the *GRAS* gene family has been reported for coriander, celery and carrot, three economically and medicinally important Apiaceae vegetables, which creates a critical research gap for comparative and functional genomics in Apiales. Here, we performed a multi-tiered bioinformatic pipeline integrating gene identification, phylogenetic classification, chromosomal mapping, gene duplication analysis, transcriptome profiling, co-expression network construction and large-scale evolutionary tracing across 406 plant genomes. In total, 87, 74 and 74 *GRAS* genes were identified from coriander, celery and carrot, respectively, which were divided into 13 subfamilies. WGD/segmental duplication drove GRAS expansion in coriander and carrot, while dispersed duplication dominated in celery. Tissue-specific expression and cross-TF regulatory networks uncovered core GRAS hub genes participating in developmental and stress pathways. Wide-range phylogeny further validated that *GRAS* genes originated from Zygnematophyceae algae and massively expanded in *Penium margaritaceum*. This study fills the research gap of GRAS family analysis in Apiaceae, provides abundant high-throughput data resources, and offers fundamental evolutionary clues for future functional verification and genetic improvement of horticultural crops.

## 1. Introduction

Transcription factors play a very important role in plant growth and response to various abiotic stresses [1,2,3,4,5,6]. Among them, GRAS transcription factors play an invaluable role in many processes of plant growth and development, such as the formation of axillary bud meristems, and root radial formation [7,8,9,10]. The GRAS name comes from the first three identified characteristic members, GAI, RGA, and SCR [11]. GRAS usually consists of 400–700 amino acids, and its C-terminal sequence is highly homologous due to five highly conserved motifs (LHR I, VHIID, LHR II, PHYRE, and SAW) [12,13,14]. Among these motifs, the VHIID motif is the most important, and there are several amino acids, such as V-valine, H-histidine, I-isoleucine, and D-aspartic acid. Of these amino acids, only histidine and aspartic acid are conserved, and the VHIID motif is present in all members of the GRAS family [12,15]. The VHIID motif is flanked by two leucine heptapeptide repeats (LHRI and LHRII), which are critical for protein-protein interactions [13]. Although the C-terminal sequences of GRAS family proteins are highly conserved, their N-terminal sequences are highly divergent [16,17]. The N-terminus of GRAS family proteins contains homopolymeric extensions of certain amino acid residues, such as proline, glutamic acid, and tyrosine, and these variable sequences determine the specificity of GRAS family protein function [16,18].

At present, a large number of *GRAS* family genes have been reported, such as the eight previously reported *GRAS* family genes, *DELLA*, *HAM*, *LISCL*, *PAT1*, *LAS*, *SCR*, *SHR*, and *SCL3*, and the functions of these *GRAS* family genes have been described in detail [19]. However, some *GRAS* family genes in grape, tomato, and other species could not be grouped with the above eight types of genes, and 5 novel types of genes appeared (GRAS8, GRASV1, GRASV2, GRASV3, and SCL26) [12,20]. As an indispensable transcription factor for plant growth and development, *GRAS* has been reported in many plants, such as *Arabidopsis* (32 *GRAS* genes), rice (57) [21], grape (52) [12], tomato (53/54) [20,22], Chinese cabbage (48) [13], *Prunus mume* (46) [23], *Fragaria vesca* (54) [24], *Setaria italica* (57) [25], *Sorghum bicolor* (81) [26], *Gossypium hirsutum* (150) [27], *Hordeum vulgare* (62) [28], *Panicum virgatum* (144) [29], *Citrus sinensis* (50) [30], *Camellia sinensis* (52) [31], *Pyrus bretschneideri* (99) [32], *Cucumis sativus* (37) [33], *Nelumbo nucifera* (38) [34], *Ipomoea trifida* (70) [35], *Glycine max* (117) [36], *Medicago sativa* (51) [37], *Capsicum annuum* (50) [38], *Juglans regia* (52) [16], *Zea mays* (86) [39], *Brassica juncea* (88) [40], *Malus domestica* (127) [41], *Litchi chinensis* (48) [42], *Solanum melongena* (48) [43], *Lagenaria siceraria* (37) [44], and *Medicago truncatula* (59) [45].

Coriander (*Coriandrum sativum*), celery (*Apium gravelolens*), and carrot (*Daucus carota*) are three of the most representative vegetable crops in the Apiaceae family. As a crucial class of transcription factors in plants, the GRAS family has not yet been systematically investigated in these three Apiaceae vegetables. The recent completion of whole-genome sequencing for these species has laid a foundational basis, enabling genome-wide studies on the characteristics, evolution, and potential functions of their *GRAS* gene families [46,47]. Coriander, commonly known as cilantro, has a mild fragrance. It is not only consumed as an edible ingredient but also recognized for its significant utility value and substantial benefits to human health [48]. Celery, cultivated worldwide, is an important vegetable crop; its parts can be used as a medicinal material to treat certain human diseases [49,50]. Carrots, rich in α-carotene and β-carotene, are also an excellent source of vitamin K and vitamin B6—nutrients that help the human body combat specific illnesses [51].

In general, these three plants serve dual purposes: they are consumed as vegetables and utilized as medicinal materials. Consequently, they are globally renowned, widely favored by people, and have become indispensable ingredients in daily diets. This study intends to conduct a comprehensive analysis of the *GRAS* gene family in these three species through in-depth bioinformatics approaches, and further explore the evolutionary imprints and origins of *GRAS* family genes across the plant kingdom. In this study, we adopted a multi-layered species selection strategy to fulfill distinct research objectives. Coriander, celery and carrot, three typical edible and medicinal Apiaceae crops, were set as core research subjects for comprehensive *GRAS* gene characterization. To conduct reliable phylogenetic comparison, we further incorporated *Arabidopsis thaliana* and grape, two well-studied model plants with abundant GRAS functional evidence, as well as lettuce, a species with close phylogenetic affinity to Apiales, forming a six-species dataset for phylogenetic tree construction. Multi-tissue RNA-seq data were only available for celery and coriander, so their transcriptomic profiles and co-expression regulatory networks were analyzed to reveal tissue-specific expression patterns. Furthermore, a total of 406 high-quality genomes spanning algae, bryophytes, ferns, gymnosperms and angiosperms were collected to systematically trace the ancient evolutionary origin of the *GRAS* gene family. Using integrated bioinformatics pipelines including gene identification, phylogenetic reconstruction, chromosomal localization, duplication type analysis, expression profiling and large-scale evolutionary comparison, we systematically uncovered the classification, expansion mechanism, tissue expression characteristics and evolutionary origin of Apiaceae *GRAS* genes, providing valuable genomic resources for subsequent functional verification and crop genetic improvement.

## 2. Materials and Methods

### 2.1. Collection of Genome Sequences and Identification of GRAS Gene Family

The sequences of celery, coriander, carrot, and lettuce were downloaded from our TVIR (http://tvir2.bio2db.com) (accessed on 28 May 2026) and TAGR database (http://tagr.bio2db.com) (accessed on 28 May 2026) [52,53,54,55]. The *A. thaliana* gene sequence was downloaded from the *Arabidopsis* Information Source (TAIR, https://www.arabidopsis.org/) (accessed on 28 May 2026) [56]. In addition, the whole-genome protein sequences of other species were downloaded from the PlantGIR database (http://plantgir.cn) (accessed on 28 May 2026) [57].

*GRAS* gene family members of all species were identified using the Pfam database integrated within the InterPro (EMBL-EBI) platform (https://www.ebi.ac.uk/interpro/) (accessed on 28 May 2026) numbered PF03514 (E-value <1 × 10^−5^) [58,59,60]. To ensure the accuracy of the results, domain validation was performed using the SMART (http://smart.embl-heidelberg.de/) (accessed on 28 May 2026) database and CDD (https://www.ncbi.nlm.nih.gov/Structure/cdd) (accessed on 28 May 2026) [61,62].

### 2.2. Chromosomal Localization, Gene Structure, and Conserved Motif Analyses

The location information of the GRAS family genes in the chromosomes of coriander, celery, and carrot was obtained from the General Feature Format (gff) file and then visualized by MapChart software (MapChart v6.5.1) [17]. The gene structure analysis, including the position of exons, introns, and untranslated regions (UTRs), was conducted using Gene Structure Displayer Server v2.0 (GSDS, http://gsds.gao-lab.org) (accessed on 28 May 2026) [63]. The conservation of amino acid sequences among carrot, celery, and coriander was performed using Multiple Expectation Maximization for Motif Elicitation (MEME, https://meme-suite.org/meme/) (accessed on 28 May 2026) [64].

### 2.3. Phylogenetic Analysis of GRAS Family Gene and Identification of Homologous Genes

The phylogenetic tree was constructed using the amino acid sequences of *GRAS* family genes. The protein sequences of *GRAS* family genes from related species were aligned using the MAFFT program (--maxiterate 1000) [65,66]. Then, the maximum likelihood tree was constructed by FastTree software (v2.2) with 1000 bootstrap replicates [67].

Orthologous and paralogous pairs in the coriander, celery, and carrot *GRAS* gene families were identified by the software OrthoMCL 2.0 (https://orthomcl.org/orthomcl/) (accessed on 28 May 2026) [68], and then the relationship between the orthologous and paralogous pairs was drawn using the Circos program (http://circos.ca/) (accessed on 28 May 2026) [69].

### 2.4. Analysis of Duplication and Loss of GRAS Family Genes During Evolution

The duplication or loss of genes in the process of evolution is assessed by reconciling the species tree and gene tree using the Notung software (v2.9) (http://amberjack.compbio.cs.cmu.edu/Notung/download.html) (accessed on 28 May 2026) [70]. First, a species tree was constructed according to the relationship among six species (coriander, celery, carrot, *Arabidopsis*, lettuce, grape), and then the species tree and gene tree were reconciled by Notung software.

### 2.5. Analysis of Collinearity and Gene Duplication Type

The collinearity of coriander, celery, and carrot *GRAS* family genes was analyzed using MCScanX software (v1.0) [71,72]. First, the whole-genome sequences of three species were aligned by the Blastp program (e-value was set to 1 × 10^−5^). Then, collinear genes were detected by MCScanX (parameter settings: −k 50, −s 5, −m 25). Finally, *GRAS* family genes located on the collinear block were extracted by a Perl script. The duplication type of *GRAS* family genes was identified using the MCScanX downstream program duplicate_gene_classifier.

### 2.6. GO Enrichment Analysis

To further elucidate the potential biological functions of differentially expressed *GRAS* genes, Gene Ontology (GO) enrichment analysis [73] was performed for *A. graveolens*, *C. sativum* and *D. carota* using the R package clusterProfiler v4.0 [74]. Custom gene-to-GO annotation files were generated by mapping whole-genome protein sequences against the eggNOG database, and all protein-coding genes with valid GO annotations in each genome were defined as the background gene universe. Over-representation analysis (ORA) based on the hypergeometric distribution was implemented via the enricher function to identify enriched GO terms, which were classified into three core ontologies: biological process (BP), cellular component (CC), and molecular function (MF). The Benjamini–Hochberg false discovery rate (FDR) method was used for multiple testing correction.

### 2.7. Tissue Differential Expression Analysis of GRAS Family Genes

To explore the expression patterns of *GRAS* family genes in coriander and celery in different tissues, we used the RNA-seq data obtained in our laboratory for analysis according to the previous reports [75,76,77]. These expression data contained three tissues (roots, petioles, and leaves) with three replications. Root, petiole and leaf tissues were collected from healthy plants at the full flowering stage under standard cultivation conditions. The expression values were normalized as reads per kilobase per million reads (RKPM). Gene expression abundances were quantified using HTSeq 2.0 [78], and differentially expressed genes (DEGs) were screened via DESeq with the adjusted *p*-value threshold set as padj < 0.05 [79]. Finally, cluster analysis was performed using TBtools (v2.0) [80].

### 2.8. Correlation and Co-Expression Network Construction

All the transcription factor families were identified using PlantTFDB according to previous reports [81,82]. The Pearson correlation coefficient (PCC) method was used to analyze the correlation within or between *GRAS* family genes and other transcription factor families according to previous reports [83,84]. It was defined as positive or negative co-expression when the PCC value of gene pairs was greater than 0.99 or lower than −0.99, respectively. Then, the interaction networks were constructed using Gephi (https://gephi.org) (accessed on 28 May 2026) software (v0.11.2) according to PCC values [83].

## 3. Results

### 3.1. Identification, Classification, and Phylogenetic Analysis of GRAS Genes

A total of 87, 74, and 74 *GRAS* genes were identified in coriander, celery, and carrot, respectively (Table S1). Additionally, 34, 72, and 53 *GRAS* genes were detected in *Arabidopsis* (*A. thaliana*), lettuce (*Lactuca sativa*), and grape (*Vitis vinifera*), respectively (Table S1). *Arabidopsis* and grape were selected as representative model species in this study, given their well-characterized *GRAS* gene families and extensive prior research. Lettuce was included due to its close phylogenetic relationship with Apiaceae species, facilitating evolutionary comparisons.

**Figure 1:**
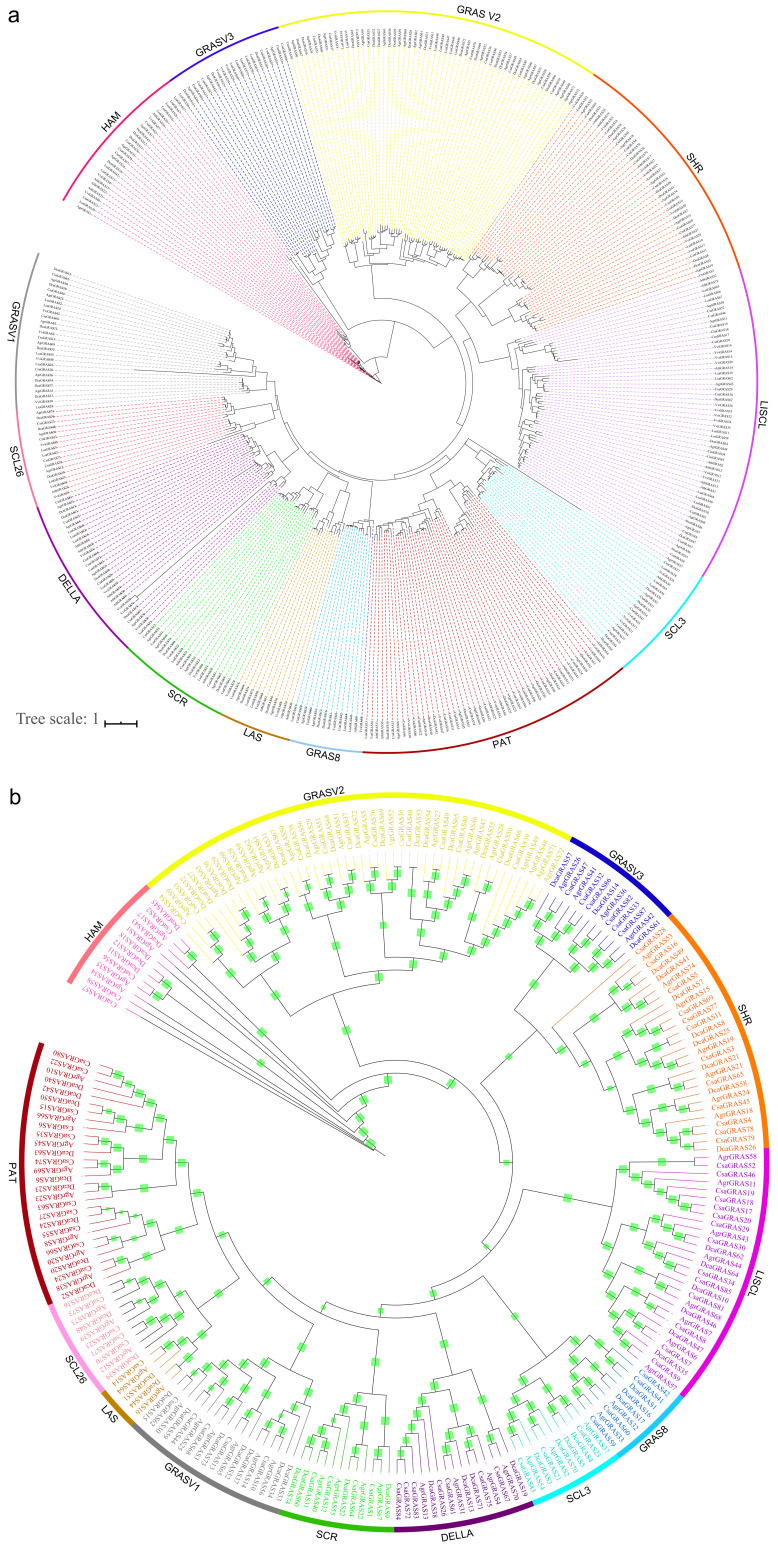
Phylogenetic analysis of *GRAS* family genes. (**a**) Phylogenetic tree of *GRAS* family genes in 3 Apiaceae (celery, carrot, and coriander), lettuce, *Arabidopsis*, and grape. (**b**) Phylogenetic tree of *GRAS* family genes in celery, carrot, and coriander. The phylogenetic tree was constructed using FastTree with maximum likelihood (ML) methods. The bootstrap value was set to 1000, and values > 40% were shown on the tree.

To investigate the evolutionary dynamics of the *GRAS* gene family, a phylogenetic tree was constructed using the *GRAS* genes identified across these six species (Figure 1a). Based on three key criteria—phylogenetic tree topology, conserved domain characteristics, and the established classification framework of GRAS family members in grape and *Arabidopsis*—the *GRAS* genes were categorized into 13 distinct subclasses, namely HAM, DELLA, LISCL, PAT, LS, SCR, SHR, SCL3, SCL26, GRASV1, GRASV2, GRASV3, and an additional subclass consistent with prior nomenclature (Figure 1a, Table S2) [12].

To further investigate the evolutionary history of the GRAS family in Apiaceae, phylogenetic trees were constructed using *GRAS* genes from coriander, celery, and carrot (Figure 1b). Consistent with grape, the three Apiaceae species were found to harbor a large number of *GRAS* genes in the PAT and LISCL subclasses (Figure 1). In contrast, a striking difference was observed in the GRASV2 subclass: grape contained the fewest GRASV2 genes, whereas the three Apiaceae species exhibited the highest gene counts in this subclass. This discrepancy is likely attributed to significant expansion of the GRASV2 subgroup driven by genome duplication events during Apiaceae evolution.

To assess the conservation of *GRAS* family genes, we analyzed their conserved motifs and gene structure. A total of 10 conserved motifs were identified, among which six (motifs 1, 2, 3, 7, 8, and 9) were ubiquitous across all *GRAS* genes (Figure S1a). These six core motifs corresponding to the characteristic GRAS domains (LHRI, VHIID, LHRII) are highly conserved across all GRAS proteins. The histidine (H) and aspartic acid (D) residues within the VHIID motif are fully conserved. However, motif absence was observed in specific subclasses: for instance, motif 5 was absent in most SCL3 subclass genes, while motifs 4, 8, and 10 were missing from GRASV3 subclass genes. The expanded PAT, LISCL and GRASV2 subfamilies possess unique motif combinations associated with their functional divergence (Figure S1a).

Gene structure analysis, conducted using the GSDS program (Figure S1b), revealed that most *GRAS* genes contained only one or two exons. Notably, two SCL3 subclass genes—*DcaGRAS3* and *DcaGRAS4*—exhibited unusual exon counts, with 16 and 10 exons, respectively.

Collectively, *GRAS* genes belonging to the same class or subclass shared similar conserved motif profiles and gene structures. These results provide strong support for the reliability of our earlier phylogenetic classification of the GRAS family.

### 3.2. Chromosomal Localization Analysis of the GRAS Gene Family

**Figure 2:**
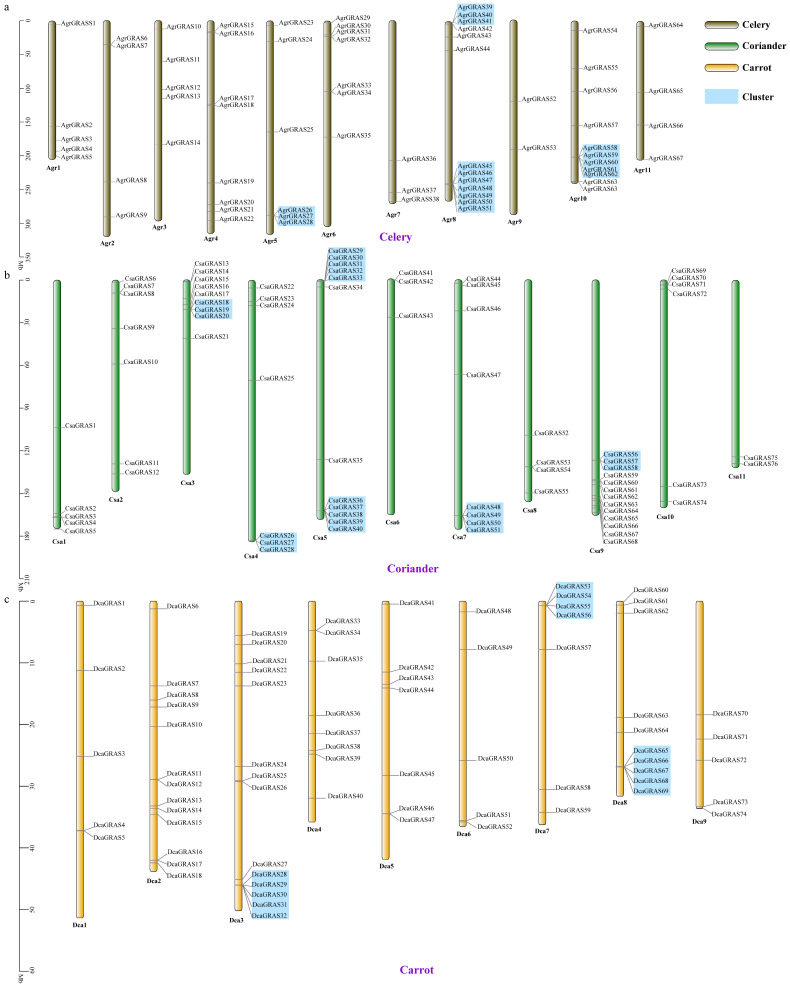
Distribution of *GRAS* family genes on each chromosome of three Apiaceae species. (**a**) celery. (**b**) coriander. (**c**) carrot.

Using chromosomal location data for the three Apiaceae vegetables, we mapped the distribution of *GRAS* family genes across their chromosomes (Figure 2).

In celery, 4 out of the 74 identified *GRAS* genes could not be assigned to any chromosome; the remaining 70 genes were unevenly distributed across 11 chromosomes (Figure 2a). Chromosome 8 harbored the highest number of *GRAS* genes (13), followed by chromosome 10 (11). In contrast, chromosomes 7 and 9 contained the fewest *GRAS* genes, with 3 and 2 genes, respectively. Additionally, four *GRAS* gene clusters—comprising 18 genes in total—were detected at the proximal ends of chromosomes 5, 8, and 10; this clustering pattern is likely a result of gene duplication events.

For coriander, the 11 unlocalized coriander *GRAS* genes are anchored to unassembled scaffold contigs that cannot be mapped to assembled chromosomes, while the remaining 76 genes were unevenly distributed across 11 chromosomes (Figure 2b). Coriander exhibited more *GRAS* gene clusters than celery: a total of 6 clusters (23 genes) were found on chromosomes 3, 4, 5, 7, and 9. Chromosome 9 had the highest *GRAS* gene count (13), whereas chromosome 11 had the lowest (2).

In carrot, all 74 *GRAS* genes were mapped to its 9 chromosomes, though their distribution was uneven (Figure 2c). Chromosomes 1 and 6 contained the fewest *GRAS* genes (5 each), while chromosomes 2 and 3 had the highest counts (13 and 14, respectively). Only 3 *GRAS* gene clusters (14 genes total) were detected in carrot, located on chromosomes 3, 7, and 8. Similar to celery and coriander, most of these clusters in carrot were positioned near chromosome ends.

Collectively, these analyses revealed that *GRAS* family genes exhibit clustered distribution patterns in coriander, celery, and carrot. This clustering is likely driven by gene duplication—an important mechanism underlying the expansion of the *GRAS* gene family in these Apiaceae vegetables.

### 3.3. Identification of Homologous Genes in the GRAS Family Across Three Apiaceae Vegetables

**Figure 3:**
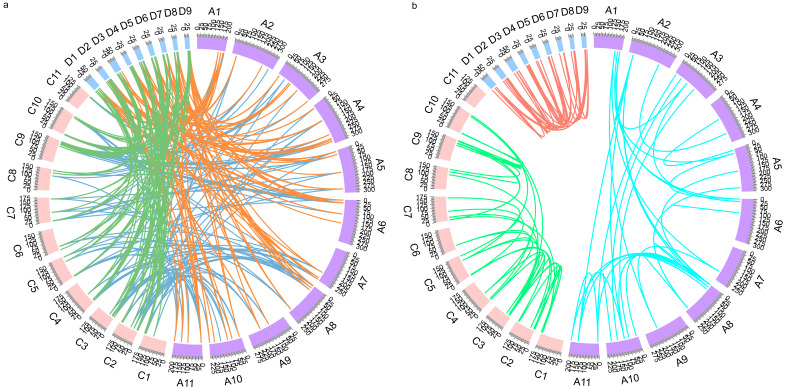
Circle plot of orthologous and paralogous *GRAS* family gene pairs among coriander, celery and carrot. (**a**) Orthologous genes. (**b**) Paralogous genes.

Homologous genes consist of two categories: orthologs (genes derived from a common ancestral gene and separated by speciation events) and paralogs (genes originating from gene duplication within a single genome). To gain deeper insights into the evolutionary origins and diversification of the GRAS family in the three Apiaceae vegetables, we systematically identified orthologous and paralogous gene pairs among them (Figure 3a, Table S3).

Orthologous gene analysis revealed 131 ortholog pairs between coriander and celery, 110 between celery and carrot, and 123 between coriander and carrot. The high number of orthologous pairs across all three species indicates that the GRAS family has remained highly conserved during the evolutionary divergence of Apiaceae, reflecting its functional importance in these plants.

In addition to orthologs, we identified paralogous gene pairs within each species: 46 in celery, 39 in coriander, and 43 in carrot (Figure 3b, Table S4). These paralogs likely arose from lineage-specific gene duplication events, which may have contributed to the functional expansion of the GRAS family in individual Apiaceae vegetables.

### 3.4. Analysis of GRAS Gene Duplication Types in Three Apiaceae Vegetables

**Figure 4:**
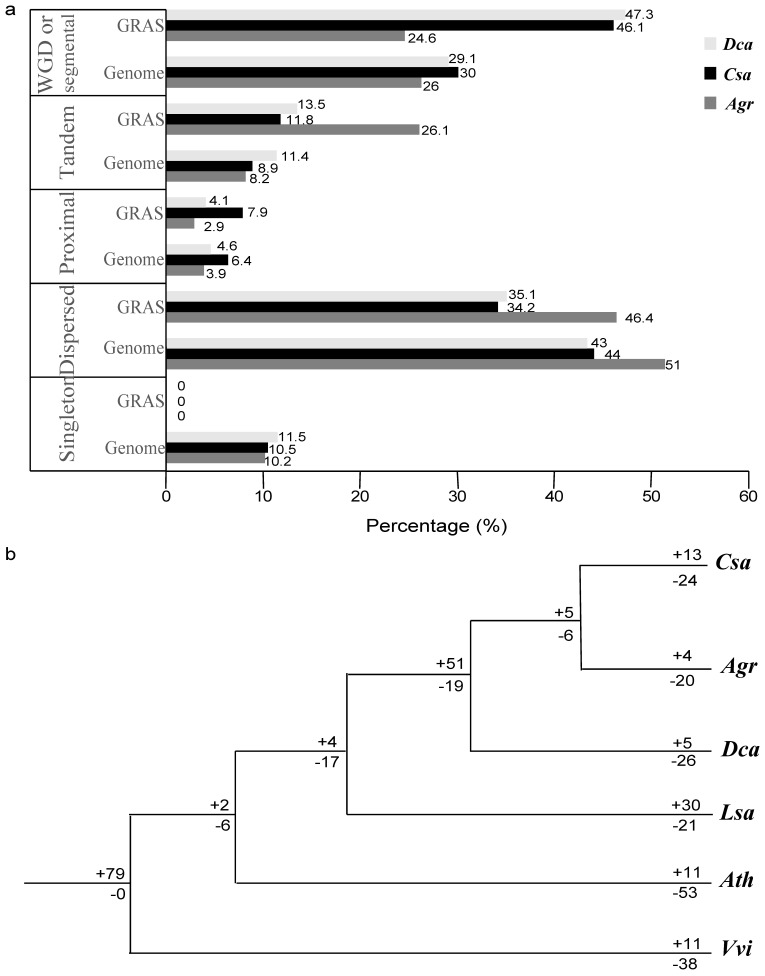
Duplication type and gene loss or duplication analysis of *GRAS* family genes. (**a**) The percentage of duplication type for *GRAS* family genes and whole-genome genes in three Apiaceae species. Dca, *D. carota* (carrot); Csa, *C. sativum* (coriander); Agr, *A. graveolens* (celery). (**b**) The duplication or loss analysis of *GRAS* family genes in three Apiaceae species and three other representative species. The “+” and “−” indicate duplication and loss, respectively. The number after “+” and “−” represents the number of genes. Lsa, *L. sativa* (lettuce); Ath, *A. thaliana* (*Arabidopsis*); Vvi, *V. vinifera* (grape).

Gene duplication is a key driver of gene family expansion. To investigate the mechanisms underlying the diversification of the GRAS family in the three Apiaceae vegetables, we analyzed the duplication types of identified *GRAS* genes using MCScanX (Figure 4a, Table 1 and Table S5). Five distinct duplication types were classified: singleton, proximal, dispersed, tandem, and whole-genome duplication/segmental duplication (WGD/Segmental).

In celery, dispersed duplication was the dominant type, accounting for 46.38% (32/69) of all *GRAS* genes. In coriander and carrot, WGD/Segmental duplication prevailed: it contributed to 46.05% (35/76) of *GRAS* genes in coriander and 47.30% (35/74) in carrot (Table 1 and Table S5). Notably, no *GRAS* genes were classified as “singleton” (genes with no detectable duplication history) across all three species. These results indicate that the expansion of the *GRAS* gene family has been shaped by distinct duplication mechanisms in celery versus coriander and carrot: dispersed duplication drove GRAS family expansion in celery, while WGD/segmental duplication played a central role in coriander and carrot.

### 3.5. Detection of GRAS Gene Family Duplications and Losses

To reconstruct the evolutionary dynamics of gains and losses in the GRAS family, we employed Notung software—a tool specialized for reconciling gene trees with species trees to infer duplication and loss events under the parsimony principle. Using our reconstructed species tree and *GRAS* gene tree, we quantified *GRAS* gene duplications and losses across the six studied species (Figure 4b).

In the common ancestor of coriander and celery, 5 *GRAS* gene duplications and 6 gene losses were detected. In the deeper common ancestor of coriander, celery, and carrot, a more pronounced expansion occurred, with 51 duplications outweighing 19 losses. Notably, coriander harbored more *GRAS* genes than celery and carrot. This disparity can be explained by two complementary patterns in gene turnover: first, coriander exhibited a higher net duplication count (+13) compared to celery (+4) and carrot (+5); second, coriander experienced fewer gene losses (−24) than carrot (−26). Among all six species, lettuce showed the most extreme duplication event, with 30 *GRAS* gene duplications—far exceeding the counts in the other five species. This dramatic expansion is consistent with prior reports of whole-genome triplication (WGT) in the lettuce genome, suggesting that WGT-driven duplication was a major force shaping the GRAS family in this lineage [85].

### 3.6. GO Pathway Enrichment Analyses of GRAS Family Genes

Gene Ontology (GO) enrichment analysis was conducted on differentially expressed *GRAS* genes from celery (*A. graveolens*), coriander (*C. sativum*) and carrot (*D. carota*) (Table S6). Across the three species, molecular function (MF) terms were predominantly enriched in transcription coregulator activity and transcription coactivator activity, which aligns with the inherent transcription factor characteristics of GRAS proteins. Cellular component (CC) enrichment terms were limited in number, mainly annotated to endosome and nuclear membrane structures with weak overall enrichment signals. Biological process (BP) exhibited highly conserved enriched pathways across all three crops, including gibberellic acid-mediated signaling (GO:0009740) and regulation of seed dormancy (GO:2000033), indicating that *GRAS* genes perform conserved biological functions in gibberellin signal transduction and seed developmental regulation. Meanwhile, distinct species-specific BP enrichment patterns were detected: *GRAS* genes from celery were significantly enriched in pathways associated with arbuscular mycorrhiza and symbiotic fungal responses; coriander *GRAS* genes were prominently enriched in processes responding to xenobiotic stimuli and photooxidative stress; carrot *GRAS* genes showed specific enrichment in salicylic acid-mediated hormone signaling pathways, which demonstrates functional divergence of *GRAS* family genes in stress adaptation among the three tested crops.

### 3.7. Differential Expression Analysis and Co-Expression Network Construction of GRAS Family Genes in Coriander and Celery

**Figure 5:**
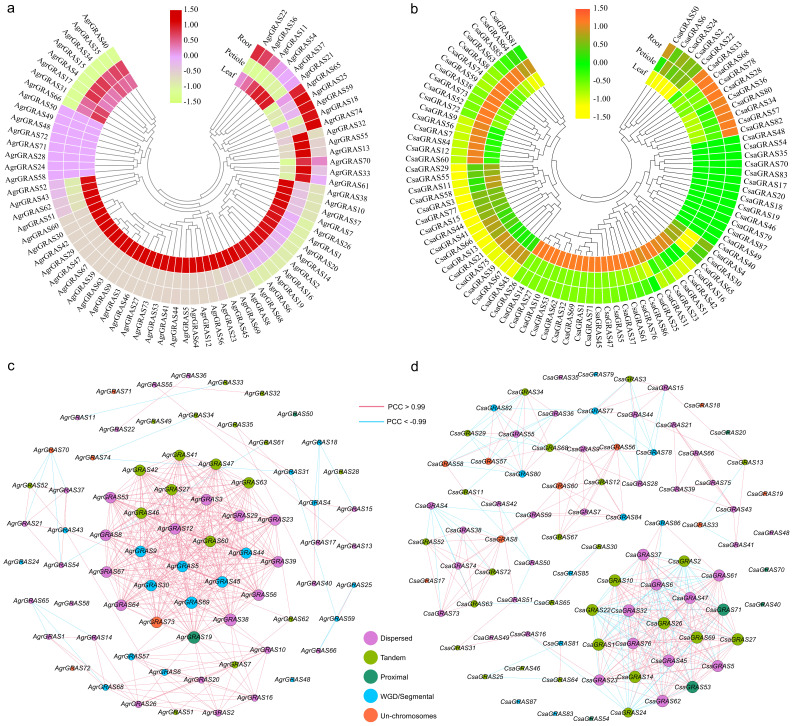
Expression heatmap and co-expression network of *GRAS* family genes in root, petiole, and leaf of celery and coriander. (**a**) Hierarchical gene expression clustering of *GRAS* family genes in celery. Expression values were normalized as RPKM and transformed by log2. (**b**) Hierarchical gene expression clustering of GRAS family genes in coriander. (**c**) The co-expression network of *GRAS* family genes in celery. The red and blue lines represent positive (PCC > 0.99) and negative (PCC < −0.99) regulation, respectively. The nodes of different colors represent each duplication type of *GRAS* family genes. (**d**) The co-expression network of *GRAS* family genes in coriander.

To explore the expression patterns of *GRAS* family genes in coriander and celery, we analyzed the gene expression differences between the two species across three tissues (root, petiole, and leaf) (Figure 5, Table S7). In celery, the gene *AgrGRAS4* exhibited significantly higher expression levels in all three tissues compared to other genes, with RPKM values of 248.09, 284.45, and 150.53 in roots, petioles, and leaves, respectively. This suggests that *AgrGRAS4* may play a crucial role in the growth and development of celery (Figure 5a, Table S7). Eight genes (*AgrGRAS58*, *AgrGRAS24*, *AgrGRAS28*, *AgrGRAS71*, *AgrGRAS72*, *AgrGRAS48*, *AgrGRAS49*, *AgrGRAS50*) showed no detectable expression in roots, petioles, or leaves, indicating that these genes might not function in these tissues at the sampling stage of this study. The celery GRAS family genes displayed distinct tissue-specific expression characteristics: 11 genes were exclusively expressed in roots, 2 genes were not expressed in roots, and 3 genes were not expressed in petioles. In the coriander *GRAS* gene family, *CsaGRAS75* had significantly higher expression levels in roots and leaves than other genes, implying that this gene may be of great importance for the growth and development of coriander (Figure 5b, Table S7). Additionally, 15 other *GRAS* genes in coriander showed no detectable expression in roots, petioles, or leaves.

In celery, the leaf vs. root (LR) comparison yielded the largest number of differentially expressed *GRAS* genes, including 13 upregulated and 8 downregulated transcripts (Table S8); the leaf vs. petiole (LP) group contained six upregulated and 11 downregulated genes, while five upregulated and nine downregulated *GRAS* genes were detected in the root vs. petiole (RP) group. Three *GRAS* genes exhibited significant expression differences across all three tissue pairs, revealing stable tissue-specific expression patterns. Most celery *GRAS* genes showed substantially higher expression levels in leaves than in roots and petioles, which was consistent with the conserved functions in gibberellin signaling and seed development revealed by GO enrichment analysis.

For coriander, the leaf vs. petiole (LP) comparison exhibited the most striking transcriptional divergence, with 11 upregulated and one downregulated *GRAS* genes (Table S9). Six upregulated and five downregulated members were found in the root vs. petiole (RP) group, and six upregulated alongside two downregulated transcripts were identified in the leaf vs. root (LR) set. Only one *GRAS* gene maintained differential expression across all three tissue comparisons, suggesting narrower tissue expression specificity in coriander relative to celery. Consistent with the prominent enrichment of pathways responding to xenobiotics and photooxidative stress in GO analysis, most coriander *GRAS* genes were highly expressed in leaf tissues.

Cross-species comparison uncovered both conserved and divergent expression features of *GRAS* family genes. The conserved trait was that the majority of differentially expressed *GRAS* genes were highly expressed in leaves, implying conserved roles of GRAS proteins in leaf development and physiological regulation. Obvious interspecific divergence was also observed: celery *GRAS* genes displayed greater expression fluctuations in root-related tissue contrasts, matching its enrichment of pathways associated with symbiotic fungal responses, whereas coriander *GRAS* genes presented more dramatic expression changes in petiole-related comparisons, reflecting lineage-specific functional adaptation to distinct growth conditions.

Based on the expression values of *GRAS* family genes in celery and coriander, we calculated the Pearson correlation coefficients (PCCs) between every pair of *GRAS* genes and constructed co-expression networks using these PCC values. After filtering, the celery co-expression network contained 66 *GRAS* family genes (nodes) with 414 connections (edges) where |PCC| > 0.99 (Figure 5c, Tables S8 and S10). Among these connections, only 17 (4.11%) were negative regulatory interactions (PCC < −0.99), while the remaining 397 were positive relationships (Figure 5c, Table S11). Furthermore, *AgrGRAS19* exhibited the highest number of connections (29), followed by *AgrGRAS38* and *AgrGRAS69* (Figure 5c and Figure S2a, Table S10). These results suggest that *GRAS* genes with more connections may serve as core regulators in the celery co-expression network.

In coriander, the co-expression network included 73 *GRAS* family genes (nodes) with 340 connections (edges) satisfying |PCC| > 0.99 (Figure 5d, Tables S12 and S13). Of these connections, 109 (32.06%) were negative regulatory interactions (PCC < −0.99), and the other 231 were positive relationships (Figure 5d and Figure S2b, Table S13). Thus, the proportion of negative connections in coriander was significantly higher than that in celery. These co-expression networks clearly reveal the regulatory relationships among *GRAS* family genes in celery and coriander, laying a foundation for future experimental studies on the functional interactions of the *GRAS* gene family.

### 3.8. Construction of Co-Expression Networks Between GRAS Family Genes and Other Transcription Factor Families in Coriander and Celery

To explore the regulatory relationships between *GRAS* family genes and other transcription factor (TF) families, we first identified all TFs from the celery and coriander genomes using the PlantTFDB database. In celery, a total of 1738 TFs belonging to 57 gene families were identified, while 2291 TFs from 58 gene families were detected in coriander. Based on the expression profiles of *GRAS* family genes and these identified TFs, we calculated the PCCs between each *GRAS* gene and every TF in both species, and subsequently constructed inter-family co-expression networks using these PCC values.

**Figure 6:**
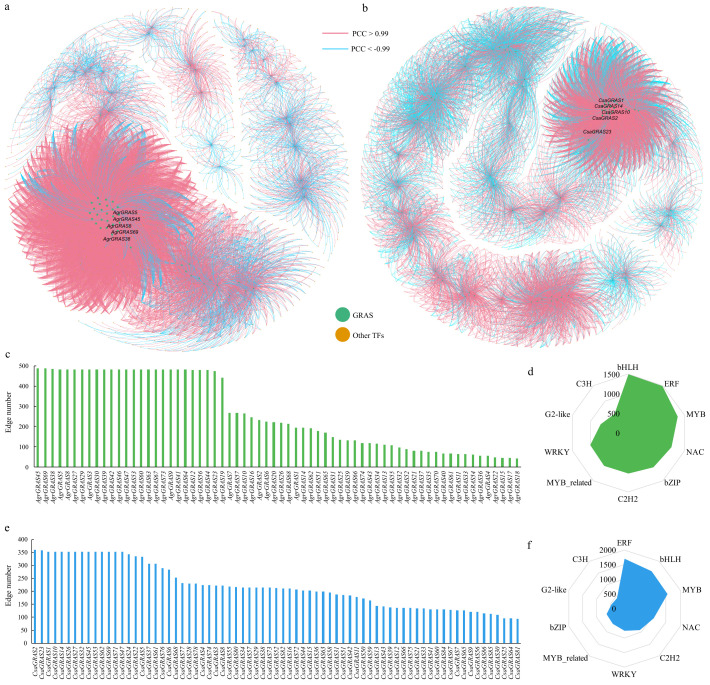
The co-expression network and statistics of *GRAS* family genes in celery and coriander. (**a**) The co-expression network of GRAS family genes and other transcription factor families in celery. The red and blue lines represent positive (PCC > 0.99) and negative (PCC < −0.99) regulation, respectively. The nodes of green and orange colors represent *GRAS* family genes and other TFs, respectively. (**b**) The co-expression network of *GRAS* family genes and other transcription factor families in coriander. (**c**) The edge number of each *GRAS* family gene in the network constructed using *GRAS* family genes and other transcription factor families in celery. (**d**) The edge number of the top 10 transcription factor families in the network constructed using *GRAS* family genes and other transcription factor families in celery. (**e**) The edge number of each *GRAS* family gene in the network constructed using *GRAS* family genes and other transcription factor families in coriander. (**f**) The edge number of the top 10 transcription factor families in the network constructed using *GRAS* family genes and other transcription factor families in coriander.

In celery, the co-expression network between *GRAS* family genes and other TF families contained 66 *GRAS* genes and 56 TF families as nodes, with a total of 17,817 connections (edges) meeting the threshold of |PCC| > 0.99 (Figure 6a, Tables S14 and S15). Among these connections, only 2637 (14.80%) represented negative regulatory interactions (PCC < −0.99), while the remaining 15,180 (85.20%) were positive relationships (Figure 6a, Table S12). For *GRAS* family genes, *AgrGRAS45* exhibited the highest number of connections (487), followed by *AgrGRAS69* and *AgrGRAS38* (Figure 6c, Table S14). Among non-GRAS TF families, the bHLH family had the most connections with *GRAS* genes (1493), followed by the ERF family (1481) and MYB family (1337) (Figure 6d, Table S14).

In coriander, the corresponding co-expression network included 73 *GRAS* genes and 57 TF families as nodes, with 16,151 connections (edges) satisfying |PCC| > 0.99 (Figure 6b, Tables S16 and S17). Of these connections, 5067 (31.37%) were negative regulatory interactions (PCC < −0.99), and the other 11,084 (68.63%) were positive relationships (Figure 6b, Table S17). For *GRAS* family genes, *CsaGRAS2* had the most connections (360), followed by *CsaGRAS23* (Figure 6e, Table S16). Among non-GRAS TF families, the ERF family formed the largest number of connections with *GRAS* genes (1698), followed by the bHLH family (1577) and MYB family (1556) (Figure 6f, Table S16).

These co-expression networks comprehensively reveal the regulatory relationships between *GRAS* family genes and other TF families in celery and coriander. Specifically, *GRAS* genes extensively interact with bHLH, ERF and MYB transcription factor families, all of which participate in hormone signal transduction and stress responses; hub *GRAS* genes with high connectivity coordinate pathways governing plant growth, development and stress adaptation. These findings lay a solid foundation for future experimental investigations into the functional interactions of *GRAS* genes with other transcription factor families.

### 3.9. Large-Scale Exploration of the Evolutionary Signatures of GRAS Family Genes

**Figure 7:**
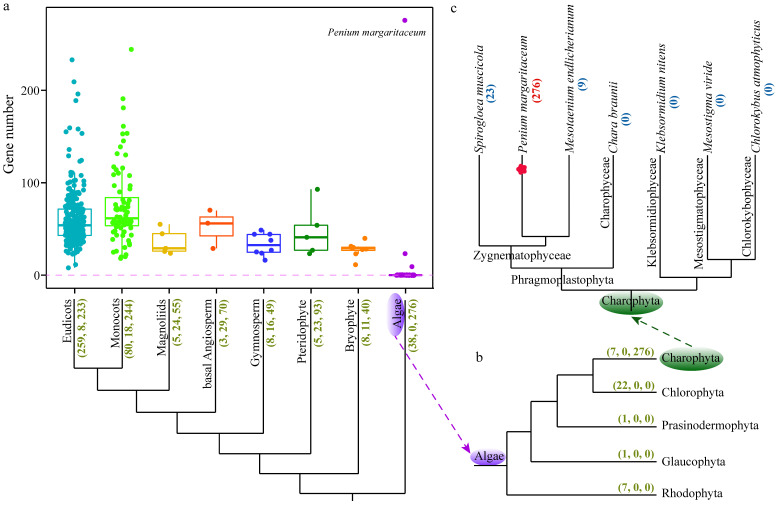
The statistics of *GRAS* family gene numbers at a large scale. (**a**) The boxplot of *GRAS* family gene number of eudicots, monocots, magnolias, Basal angiosperms, gymnosperms, ferns, mosses, and algae. The first number in parentheses represents the number of species in the classification. The second and third represent the minimum and maximum *GRAS* genes contained in the species. (**b**) The statistics of *GRAS* family gene number of different taxonomies of Algae. (**c**) The statistics of *GRAS* family gene number of different taxonomies of Charophyta. The number in parentheses represents the number of *GRAS* genes in the related species.

A total of 23,240 *GRAS* family genes were identified across 406 species with fully sequenced and well-annotated genomes (Figure 7a, Table S18). These species contained 259 eudicots, 80 monocots, 5 magnoliids, 3 basal angiosperms, 8 gymnosperms, 5 ferns, 8 mosses, and 38 algae (Figure 7a, Table S18). All surveyed higher plants contained *GRAS* genes: the eudicot *Amaranthus hypochondriacus* had the fewest (8 *GRAS* genes), while the monocot *Dendrocalamus latiflorus* had the most (244 *GRAS* genes). *GRAS* gene numbers ranged from 8 to 233 in eudicots and 18 to 244 in monocots, with most monocots and eudicots harboring more *GRAS* genes than other taxonomic groups. Notably, *GRAS* gene counts varied drastically among the 38 lower plant algae examined (Figure 7b, Table S18). No *GRAS* family genes were detected in 35 algae, whereas *Penium margaritaceum* contained 276 *GRAS* genes—more than any of the other 405 species in this study (Figure 7a,b, Table S18).

Further analysis of *GRAS* genes in the 38 algae revealed their presence exclusively in Charophyta; no *GRAS* genes were found in the 31 more basal algae (Figure 7b, Table S18). Among the 7 Charophyta species, *GRAS* genes were detected in 3 species of Zygnematophyceae, but absent from 4 species belonging to Charophyceae and other more basal families within Charophyta (Figure 7c, Table S18). These results suggest that GRAS family genes likely originated in Zygnematophyceae and underwent significant expansion in *P. margaritaceum* during evolutionary progression.

**Figure 8:**
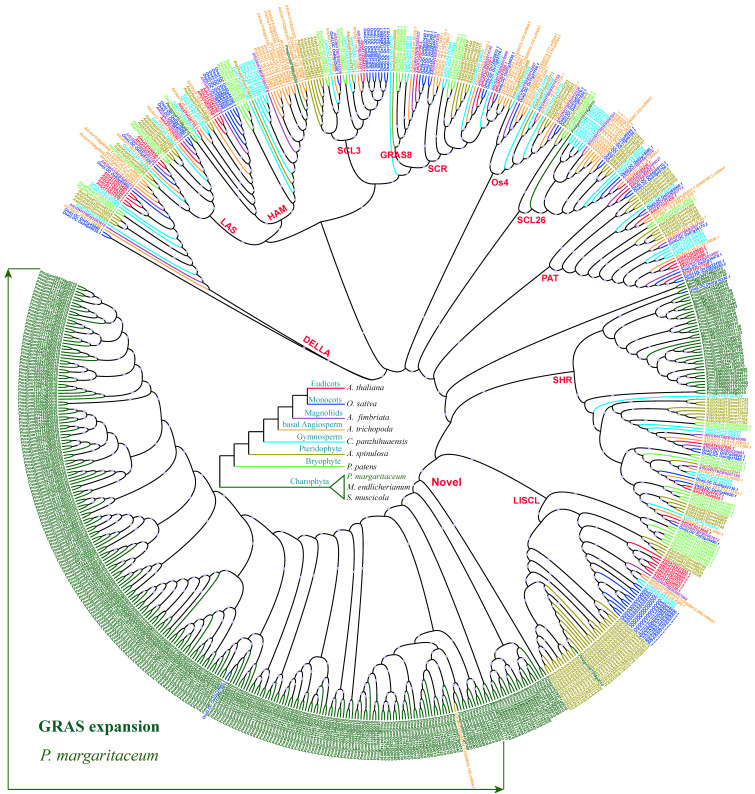
Phylogenetic tree of *GRAS* family genes in 7 selected typical species that can represent evolutionary branches and 3 Charophyta species. The phylogenetic tree was constructed using FastTree with ML methods. The bootstrap value was set to 1000, and values > 40% were shown on the tree.

To further investigate the evolutionary origin and diversification of *GRAS* genes, we selected 7 representative species covering major evolutionary lineages, combined with the 3 Charophyta species harboring *GRAS* genes, to construct a phylogenetic tree (Figure 8). The selected representative species included the eudicot model *A. thaliana*, the monocot model *Oryza sativa*, the magnoliid *Aristolochia fimbriata*, the basal angiosperm *Amborella trichopoda*, the gymnosperm *Cycas panzhihuaensis*, the fern *Alsophila spinulosa*, and the bryophyte *Physcomitrella patens*. Based on previous GRAS classification systems and the topology of our phylogenetic tree, we divided the GRAS family into 12 groups. Among these, the HAM, SHR, LISCL, and CHARO groups all contained Charophyta-derived *GRAS* genes, while the DELLA, LAS, SCL3, GRAS8, SCR, OS4, SCL26, and PAT8 groups lacked Charophyta members. Except for the OS4 group, the other 8 groups all included *GRAS* genes from the bryophyte *P. patens*, indicating that these groups originated in bryophytes. The OS4 group emerged later, first appearing in ferns, and was not represented in the eudicot *A. thaliana*. For Charophyta *GRAS* genes, the vast majority belonged to the SHR and CHARO groups. Notably, the CHARO group is a novel subgroup identified and named in this study, which has not been reported previously. Almost all members of the CHARO group were derived from the lower plant Charophyta, and this group played a crucial role in the expansion of the *GRAS* gene family in *P. margaritaceum*.

## 4. Discussion

*GRAS* family genes are widely distributed across most plant lineages and play pivotal roles in regulating various aspects of plant growth and development [8,86]. Accumulated functional studies illustrate that GRAS proteins coordinate stress adaptation via cross-talk with multiple stress-related transcription factors and hormone pathways. In *A. thaliana*, the functional characterization of most GRAS family members has been well established. For instance, the *Arabidopsis GRAS* gene *AtSCL14* (*At1g07530*) is essential for activating stress-inducible promoters, and its overexpression or functional activation confers enhanced tolerance to toxic concentrations of 2,4,6-triiodobenzoic acid and the chemical isonicotinic acid [87]. Similarly, the GRAS transcription factor PAT1 (*VIT_219s0014g04940*) from grapevine regulates jasmonic acid biosynthesis in response to cold stress, a function conserved in *Arabidopsis* [88,89]. These well-characterized *GRAS* genes from *Arabidopsis* and grapevine provide valuable references for inferring the functions of GRAS family members in Apiaceae through bioinformatics analysis. Phylogenetic analysis revealed that grapevine *PAT1* (*VviGRAS46*), a key regulator of cold stress responses, clusters closely with *CsaGRAS63*, *AgrGRAS23*, and *DcaGRAS23* from Apiaceae (Figure 1a). Based on this evolutionary affinity, we hypothesize that these three Apiaceae *GRAS* genes may be involved in mediating cold stress tolerance. Similarly, Apiaceae *GRAS* genes classified into the LISCL subfamily form a monophyletic clade with *AtSCL14* (Figure 1a), a gene implicated in tolerance to toxic compounds. Thus, it is reasonable to speculate that most LISCL subfamily genes in Apiaceae might contribute to plant tolerance against toxic substances. However, the precise biological functions of these Apiaceae *GRAS* genes, including their regulatory mechanisms and physiological roles, require further experimental validation in future studies.

Our analysis revealed that most duplicated *GRAS* family genes exhibit conserved expression patterns across different tissues. However, a subset of duplicated genes showed distinct expression divergence. For example, in celery, three duplicated genes—*AgrGRAS26*, *AgrGRAS27*, and *AgrGRAS28*—displayed notably different expression profiles across the three tested tissues (Figure 5a). Similarly, within another duplicated gene set, *AgrGRAS39* and *AgrGRAS41* shared similar expression patterns, while both differed significantly from the third duplicated paralog, *AgrGRAS40* (Figure 5a). This observed expression divergence among duplicated *GRAS* genes suggests that functional differentiation of these paralogs occurred during celery evolution, which aligns with previous reports documenting functional divergence of duplicated genes in plants [90,91]. According to established evolutionary models, duplicated genes typically undergo one of several fates, including subfunctionalization, neofunctionalization, functional conservation, or specialization [1,90,92,93]. Collectively, the expression patterns of duplicated *GRAS* genes identified in this study provide a critical foundation for subsequent functional characterization of these gene families in Apiaceae.

Previously, the *GRAS* gene family has been extensively characterized in a wide range of plant species; however, no systematic investigation of this family has been conducted in Apiaceae. The recent release of genome sequences for carrot, celery, and coriander has greatly facilitated the present study [46,47,75]. Here, we identified 74, 87, and 74 *GRAS* genes in the genomes of celery, coriander, and carrot, respectively. For comparative analysis with these three Apiaceae species, we also identified 34, 72, and 53 *GRAS* genes in *A. thaliana*, lettuce, and grapevine genomes, respectively.

To explore the evolutionary relationships of *GRAS* genes, we constructed a phylogenetic tree using GRAS protein sequences from coriander, celery, carrot, lettuce, *Arabidopsis*, and grapevine. Analyses of conserved motifs and gene structures revealed that *GRAS* family genes within the same group or subgroup shared similar structural features—a finding consistent with previous reports on *GRAS* gene families in other plants [94,95,96]. Furthermore, we found that whole-genome duplication/segmental duplication (WGD/Segmental) was the primary mode of gene duplication for *GRAS* genes in coriander and carrot, whereas dispersed duplication was the dominant type in celery.

To date, only a limited number of gene family studies have been reported in Apiaceae species [1,90,97]. This study represents the first systematic analysis of the *GRAS* gene family across three Apiaceae vegetables. Investigating these *GRAS* family genes will facilitate the understanding of the molecular genetic regulatory mechanisms underlying the genetic improvement of Apiaceae crops, while also providing functional gene resources for gene editing and transgenic research. Furthermore, this work enhances our insights into the impacts of gene duplication and loss events during the evolutionary process of Apiaceae species. Overall, our study offers valuable resources for future investigations on the evolution and functional characterization of the *GRAS* gene family in Apiaceae vegetables.

## 5. Conclusions

In the present study, we systematically identified and characterized the GRAS transcription factor family in three economically valuable Apiaceae vegetables: coriander (*C. sativum*), celery (*A. graveolens*), and carrot (*D. carota*). In total, 87, 74, and 74 *GRAS* genes were retrieved from their respective genomes, and all 235 GRAS members were divided into 13 distinct subclades based on phylogenetic topology, conserved motif composition, and gene structural features. Chromosomal mapping revealed an obvious clustered distribution of *GRAS* genes across the chromosomes of the three species, and 11 unanchored coriander *GRAS* genes were proven to be located on unassembled genome scaffolds that cannot be assigned to assembled chromosomes. Inter-species orthologous gene pairs and intra-species paralogs were extensively screened, demonstrating that GRAS families remain highly conserved during the speciation of Apiaceae crops yet underwent lineage-specific expansion via distinct duplication mechanisms. WGD/segmental duplication acted as the primary driving force for GRAS expansion in coriander and carrot, while dispersed duplication dominated the amplification of celery *GRAS* genes; across all three vegetables, gene duplication events far outnumbered gene loss events over evolutionary time.

Tissue transcriptome profiling uncovered widespread expression of *GRAS* genes in roots, petioles and leaves, with a subset of members showing striking tissue-specific expression patterns. Core hub genes such as *AgrGRAS4* in celery and *CsaGRAS75* in coriander exhibited extremely high transcript abundance in multiple tissues, implying their central roles in plant growth and development. We further constructed two layers of co-expression networks for celery and coriander: intra-family regulatory networks among GRAS members, and inter-family interaction networks linking GRAS proteins to bHLH, ERF, MYB and other key stress-related transcription factors. These network datasets provide clear clues for exploring how GRAS proteins coordinate plant stress adaptation and developmental regulation.

By leveraging genome resources from 406 plant species covering algae, bryophytes, ferns, gymnosperms and angiosperms, we traced the deep evolutionary origin of the *GRAS* gene family. Phylogenetic evidence strongly supports that *GRAS* genes first emerged in Zygnematophyceae charophyte algae and experienced massive independent expansion in *P. margaritaceum*. A novel CHARO subfamily unique to early charophyte algae was identified in this work, which contributes to the huge *GRAS* gene repertoire of this alga. Several GRAS subclades, including SHR, HAM and LISCL, originated in basal streptophytes and were retained throughout plant terrestrialization, while subgroups such as OS4 arose later in fern lineages.

Collectively, this work represents the first comprehensive genome-wide investigation of the GRAS family across three typical Apiaceae medicinal and edible vegetables. Our multi-dimensional results clarify the classification, chromosomal distribution, duplication modes, tissue expression profiles, co-regulatory networks and deep evolutionary trajectory of plant GRAS transcription factors. The abundant genomic resources and evolutionary evidence generated here lay a solid foundation for subsequent gene functional verification, genetic improvement and comparative genomic research of *GRAS* genes in Apiaceae and other crop species.

## Figures and Tables

**Table 1:** The identification of duplicated types for *GRAS* family genes and all genes in *A. graveolens* (celery), *C. sativum* (coriander), and *D. carota* (carrot).

Species	Singleton		Dispersed		Proximal		Tandem		WGD or Segmental			Total	
	All	GRAS	All	GRAS	All	GRAS	All	GRAS	All	GRAS	Percentage	All	GRAS
Celery	3028	0	15,258	32	1167	2	2426	18	7787	17	24.6%	29,666	69
Coriander	3577	0	14,963	26	2161	6	3032	9	10,200	35	46.1%	33,933	76
Carrot	3543	0	13,378	26	1428	3	3501	10	8974	35	47.3%	30,824	74

## Data Availability

The expression datasets analyzed in this study are available in the Beijing Institute of Genomics (BIG) Data Center under accession numbers CRA001996 and CRA001658, which are publicly accessible at http://bigd.big.ac.cn/gsa (accessed on 28 May 2026). All materials and related data in this study are provided in the Supplementary Materials.

## Supplementary Materials

The following supporting information can be downloaded at: https://www.mdpi.com/article/10.3390/life16071113/s1.
