# Supplementary material for: Genome-Wide Characterization of the GRAS Gene Family in Three Apiaceae Vegetables with Evolutionary Implications Across Representative Plants
Source: Life (Basel). 2026 Jul 3;16(7):1113. doi: 10.3390/life16071113 (PMC13412788; doi:10.3390/life16071113)
Supplement: Supplementary file 1 [file life-16-01113-s001.zip › Supplementary Figures S1 and S2.pdf]

Supplementary Figures S1 and S2

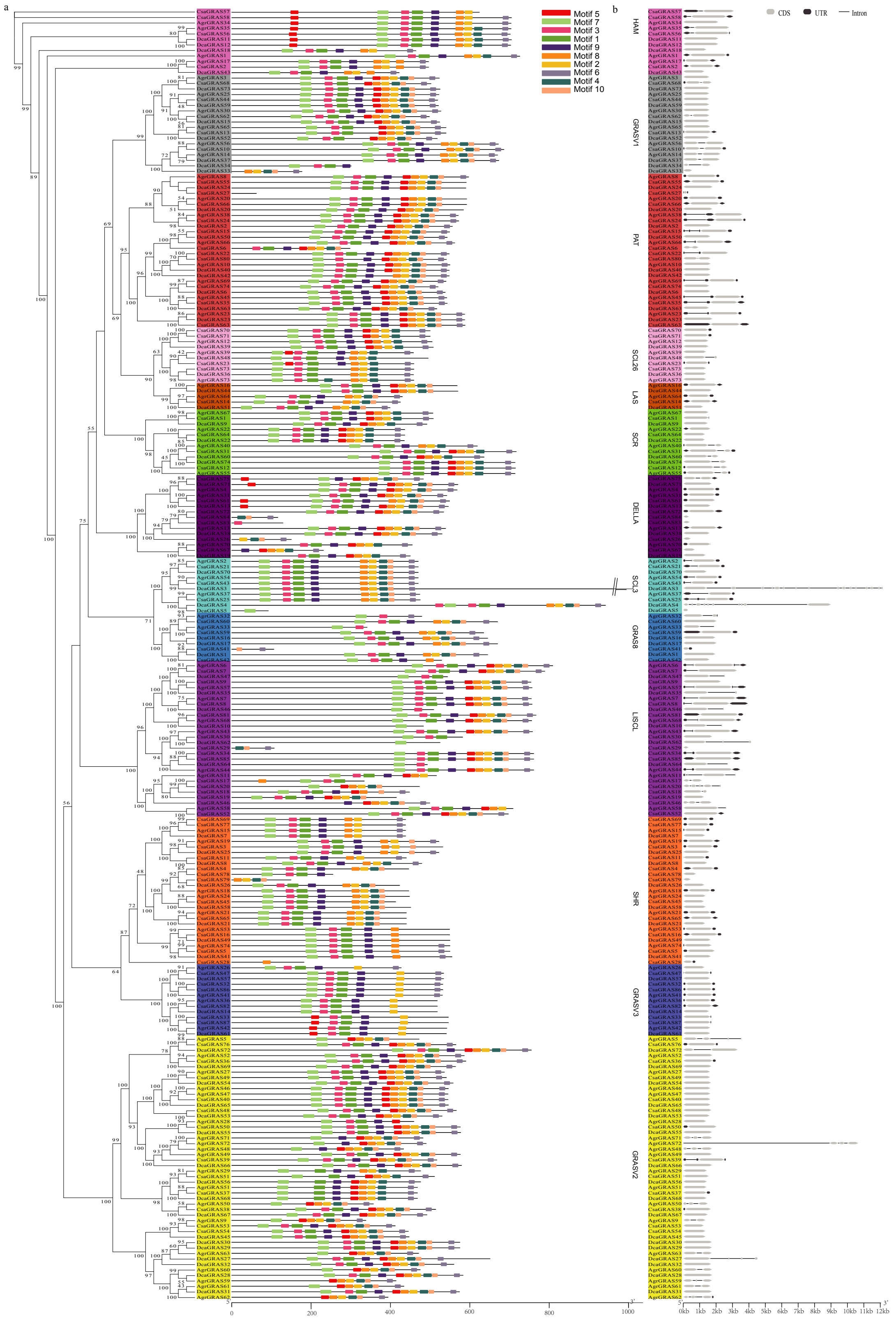

Figure S1. Conserved motif and gene structure analysis of GRAS gene family in three Apiaceae species. (a) Conserved motif. (b) Gene structure.

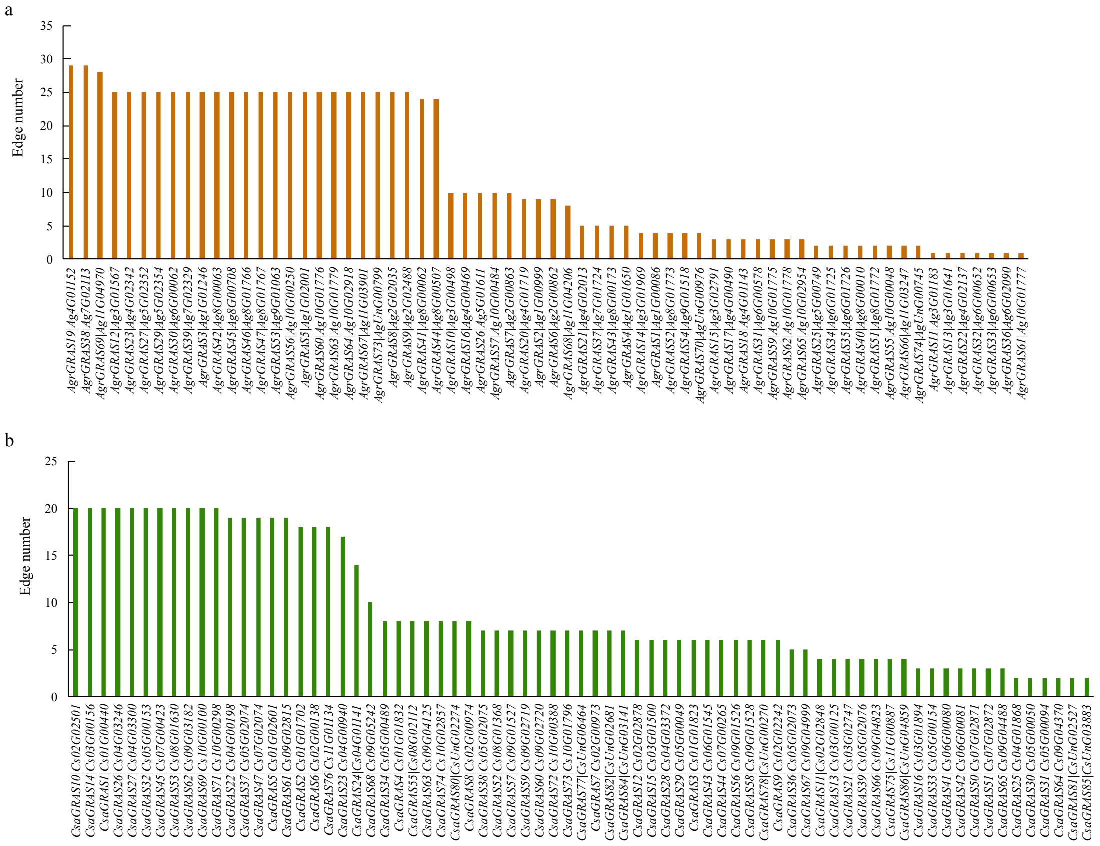

Figure S2. The edge number of each gene in the network constructed using GRAS family genes. (a) Celery. (b) Coriander.
